# Early Revision Rates and Clinical Outcomes of Two Cementless Cruciate-Retaining Total Knee Arthroplasty Designs at Two-Year Follow-Up: A Propensity Score-Adjusted Comparative Analysis

**DOI:** 10.3390/medicina62101919

**Published:** 2026-10-05

**Authors:** Olivia Schaffer, Mitchell Kennedy, Andrew Li, David Novikov, Morteza Meftah, Joshua C. Rozell, Ran Schwarzkopf

**Affiliations:** Department of Orthopedic Surgery, NYU Langone Health, New York, NY 10016, USA; olivia.schaffer@nyulangone.org (O.S.); mitchell.kennedy@nyulangone.org (M.K.); david.novikov@nyulangone.org (D.N.); morteza.meftah@nyulangone.org (M.M.); joshua.rozell@nyulangone.org (J.C.R.)

**Keywords:** cementless fixation, total knee arthroplasty, patient-reported outcomes, osseointegration, porous-coated cementless implants

## Abstract

*Background and Objectives:* Cementless total knee arthroplasty (TKA) has gained popularity but comparative outcome data across implant systems remain limited. This study compared survivorship and clinical outcomes of two cementless cruciate-retaining TKA implants from different manufacturers. *Materials and Methods:* We performed a retrospective, single-institution review of 220 patients who had a cementless cruciate-retaining TKA implant between 2019 and 2024; 110 patients received Implant A and 110 patients received Implant B. Baseline variables included demographics, comorbidities, and preoperative Knee Injury and Osteoarthritis Outcome Score for Joint Replacement (KOOS, JR). Clinical outcomes included 90-day readmission, all-time revision, reoperation, manipulation under anesthesia, and postoperative KOOS, JR scores. Propensity score-based overlap weighting was used to create a balanced pseudo-population for adjusted analyses. Adjusted outcomes included weighted logistic regression for two-year revision and a weighted Cox proportional hazards model for time to revision. *Results:* Crude revision rates were low and not significantly different (A: 0.9 versus B: 2.7%, *p* = 0.622). Overlap weighting achieved balance across all measured baseline covariates. Unadjusted rates of 90-day readmission, reoperation, and manipulation under anesthesia were similar between groups. In adjusted analyses, no statistically significant difference was observed for two-year revision in Implant B vs. A cohorts (OR 2.17, 95% CI 0.10 to 157, *p* = 0.621) or time to revision (HR 2.22, 95% CI 0.18 to 27.09, *p* = 0.533). *Conclusions:* Both cementless cruciate-retaining TKA systems demonstrated low early revision rates in this cohort. Propensity score-adjusted analyses reduced measured baseline imbalances and did not identify a significant difference in revision risk between implants; however, interpretation is limited by sparse revision events, potential residual confounding, and the retrospective observational design.

## 1. Introduction

Total knee arthroplasty (TKA) is an effective treatment for end-stage knee osteoarthritis, providing reliable pain relief and functional improvement for most patients [1,2,3]. Cemented fixation using polymethylmethacrylate has historically been the predominant method in the United States due to its immediate stability, technical reliability, and excellent long-term survivorship [4,5,6,7]. While advantageous for older and less active patients, cemented fixation can degrade over time, potentially leading to implant loosening and cement debris-related osteolysis, particularly in younger, more active, or obese patients who subject the bone–implant interface to higher mechanical stresses [5,8,9,10].

Cementless fixation offers a biologic alternative that avoids cement-related complications and achieves osseointegration, which can potentially provide more durable long-term fixation compared to the bone–cement interface [11]. Early cementless TKA designs were associated with high failure rates due to inadequate porous coatings and insufficient support for bone ingrowth, leading to osteolysis, component subsidence, and high rates of aseptic loosening [12,13,14,15]. Advancements in implant technology have allowed modern cementless implants to incorporate highly porous metals, optimized surface topography, and circumferential porous coatings designed to enhance osseointegration and fixation stability [5,11,16]. Contemporary studies suggest that these designs demonstrate survivorship comparable to or exceeding that of cemented implants, particularly in younger patient populations, and their utilization has increased correspondingly in recent years [17,18,19,20,21,22].

Despite the increasing use of cementless TKA, multiple contemporary implant systems utilizing distinct porous materials and surface technologies are currently available. Differences in porous architecture, surface coatings, and fixation design may influence osseointegration and long-term fixation reliability. However, direct comparative clinical data between modern cementless TKA systems remain limited. The purpose of this study was to compare survivorship, clinical outcomes, patient-reported outcomes (PROMs), and propensity score-based weighted outcomes between two widely used contemporary cementless cruciate-retaining implants that employ different porous fixation technologies.

## 2. Materials and Methods

### 2.1. Study Design 

A retrospective review of patients who underwent primary total knee arthroplasty between 2019 and 2024 was performed at a single institution. Patients were treated with one of two cementless cruciate-retaining TKA systems, the LEGION CONCELOC Knee System (Implant A; Smith & Nephew, Memphis, TN, USA) or the Triathlon Cementless Knee System (Implant B; Stryker, Mahwah, NJ, USA). 

Patients were eligible for inclusion if they underwent primary cementless TKA using either implant system and had a follow-up visit at two years. Exclusion criteria included revision TKA, simultaneous bilateral TKA, or age less than 18 years. Demographic characteristics, perioperative variables, and clinical outcomes were collected from the electronic medical record. Patients were evaluated at routine postoperative follow-up visits scheduled for two weeks, three months, 12 months, and 24 months after surgery, with prespecified allowable visit windows for each timepoint; however, only the date of most recent follow-up was recorded. Postoperative follow-up windows were as follows: up to 42 days for two-week, 43–180 days for three-month, 181–540 days for 12-month, and 541–913 days for 24-month follow-up, respectively. Implant survivorship was assessed for the entire cohort, with revision defined as any reoperation involving exchange or removal of the femoral or tibial components. 

Preoperatively, target limb alignment was determined based on surgeon preference, and coronal plane alignment of the knee (CPAK) morphotype was not routinely collected. Bone resection technique was determined according to implant: 89.1% of Implant A cases involved computer assistance, two (1.8%) used robotic assistance, and ten cases (9.1%) did not have any robotic assistance. By contrast, all surgeries involving Implant B involved robotic assistance. Perioperative antibiotic prophylaxis was administered as weight-based intravenous cefazolin in 50 milliliters of 5% dextrose at least 15 min prior to incision. Antibiotic treatment was continued for the first 24 h after surgery, aligning with contemporary recommendations [23,24]. Pneumatic tourniquet use was not routine for all patients and was determined according to individual surgeon preference. When used, the tourniquet pressure was typically 225 mmHg; tourniquet duration was not standardized across cases. Closed-suction drainage was not used in our cohort.

Patients in the Implant A cohort (mean age, 57.7 years; range, 24 to 74 years) were younger than those in the Implant B cohort (mean age, 63.2 years; range, 41 to 85 years) and had a higher mean BMI (Implant A 34.3; range, 21.1 to 58.4; Implant B 32.2; range, 21.0 to 50.0). Sex distribution, race, and American Society of Anesthesiologists (ASA) classification were similar between groups, while smoking status differed with more never-smokers in the Implant A cohort and more former smokers in the Implant B cohort (Table 1, *p* = 0.050). There were 16 surgeons included in the study, with eight using Implant A only, four using Implant B only, and four using both implants. 

### 2.2. Implant Systems

**Figure 1:**
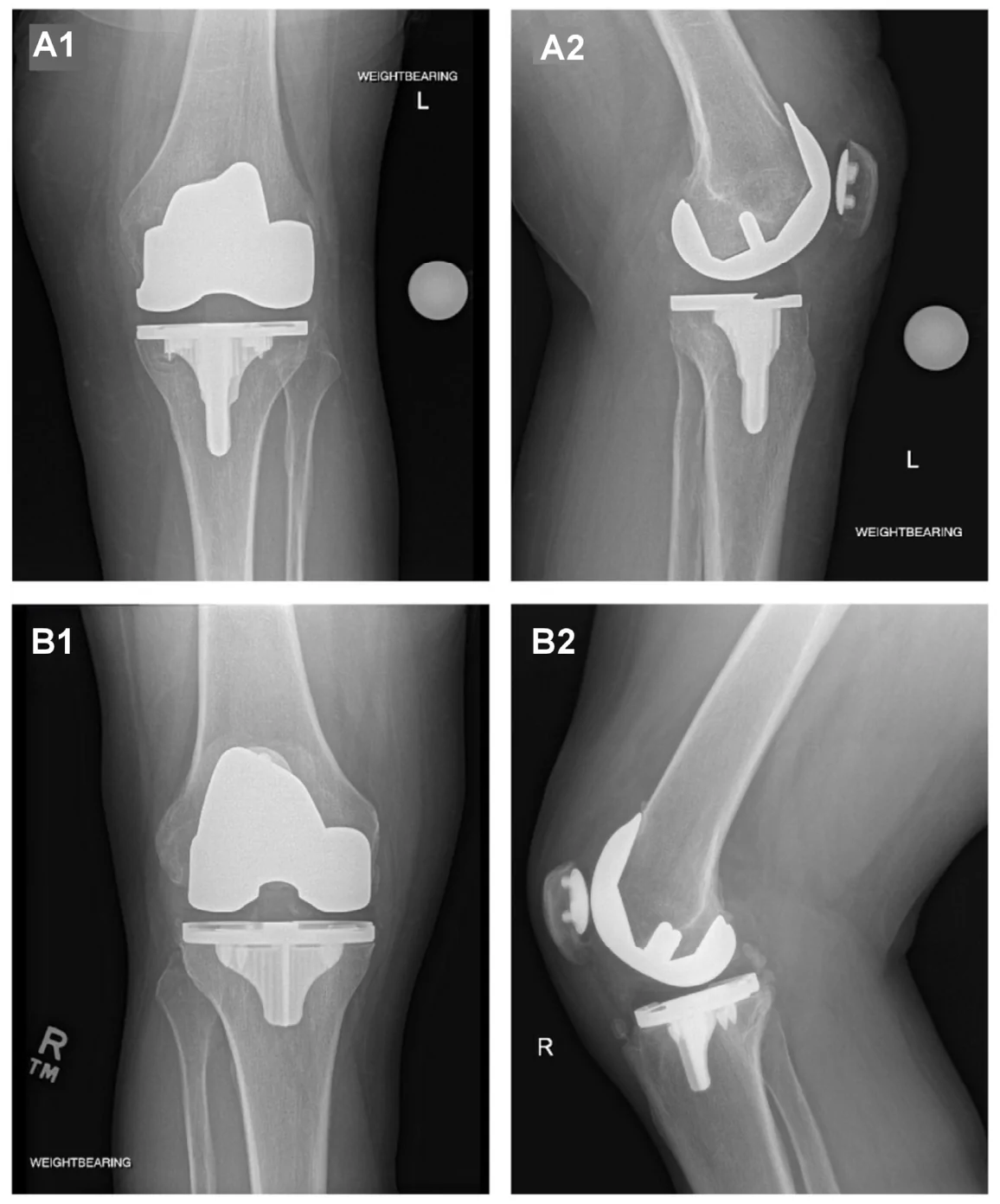
Representative radiographs of implants (**A**, CONCELOC, Smith & Nephew) and (**B**, Triathlon, Stryker). Antero-posterior (**1**, **left**) and lateral (**2**, **right**) standing radiographs of implants (**A**) (**above**) and (**B**) (**below**). Implant A: a 61-year-old woman; left total knee arthroplasty with patellar resurfacing. Implant B: a 54-year-old man; right total knee arthroplasty with patellar resurfacing.

Both investigated implants are contemporary cementless TKA systems manufactured using additive manufacturing and designed to achieve biologic fixation through porous metal surfaces that facilitate bone ingrowth (Figure 1).

**Figure 2:**
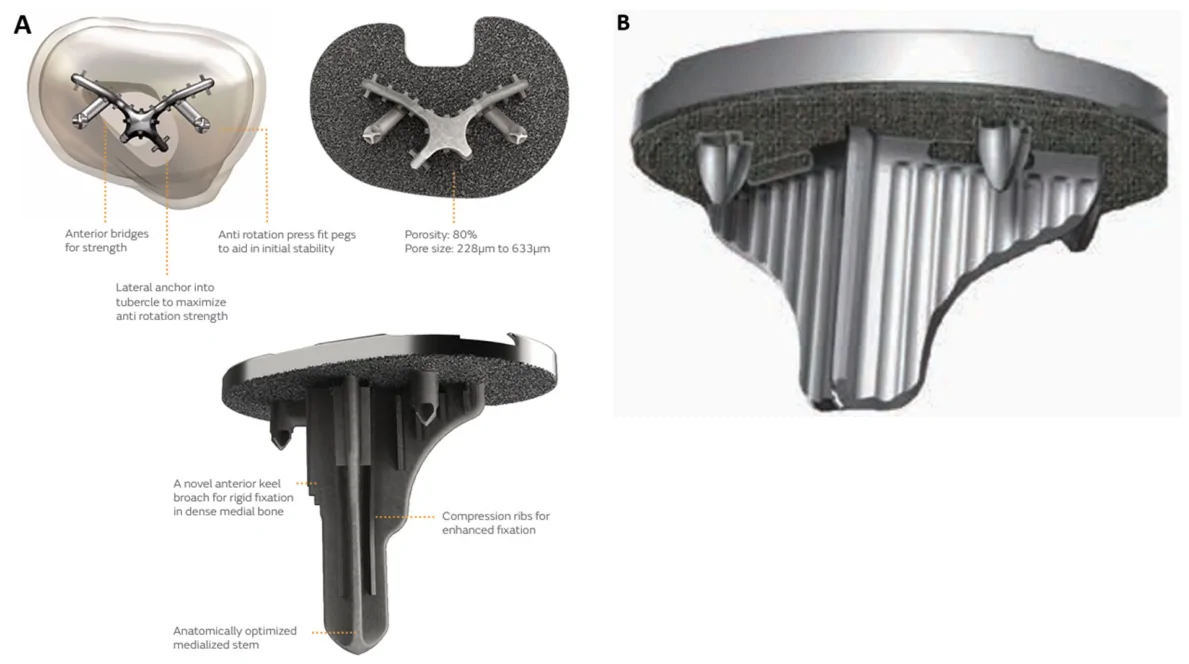
Representative illustrations of the tibial baseplates of implants (**A**,**B**).

Implant A utilizes an advanced porous titanium (CONCELOC, Smith & Nephew, Memphis, TN, USA), a manufactured porous titanium alloy designed to mimic approximate cancellous bone structure (Figure 2). The tibial component consists of a porous titanium alloy baseplate incorporating an anatomically optimized, medialized keel with anti-rotation press fit pegs to aid in initial stability. It also incorporates a lateral anchor which is intended to enhance rotational stability. The tibia also consists of an anatomic footprint and a novel anterior keel broach for rigid fixation in dense medial bone to maximize anti rotation strength. Femoral components are available in cobalt–chromium and articulate with a highly cross-linked polyethylene insert. The system also includes a cementless patellar component manufactured using additive porous titanium technology designed to support biologic fixation [25]. Implant B similarly incorporates a highly porous titanium fixation surface intended to support biologic ingrowth. The tibial baseplate features a porous titanium coating with a central keel and peripheral cruciform pegs for initial fixation (Triathlon, Stryker, Mahwah, NJ, USA). Additionally, porous structures are positioned strategically to avoid stress shielding in distal areas. The femoral component is composed of a cobalt–chromium alloy with a porous fixation surface and articulates with a highly cross-linked polyethylene insert [26].

### 2.3. Data Collection and Outcome Measures 

Patient data were abstracted from the institutional electronic medical record (Epic Caboodle Version 15; Epic Systems Corporation, Verona, WI, USA). Collected demographic variables included age at surgery, sex, race, body mass index (BMI), smoking status, insurance type, Charlson Comorbidity Index (CCI), and American Society of Anesthesiologists (ASA) classification. Perioperative variables included operative time, anesthesia type, operative laterality, patellar fixation method, patellar resurfacing status, and femoral coating type.

The primary outcome was implant survivorship, defined as freedom from all-cause revision surgery involving the femoral or tibial components. Indications for revision were recorded for all revision events. Secondary outcomes included hospital length of stay (LOS), discharge disposition, 90-day emergency department visits, 90-day hospital readmissions, and manipulations under anesthesia (MUAs). Functional outcomes were assessed using the Knee Injury and Osteoarthritis Outcome Score for Joint Replacement (KOOS JR), collected preoperatively and at two weeks, three months, one year, and two years postoperatively. Achievement of the minimum clinically important difference (MCID) in KOOS, JR at one year was determined by whether patients had an improvement from their preoperative score by 14 [27]. 

### 2.4. Data Analysis

Descriptive statistics were calculated as means with ranges for continuous variables and as counts with percentages for categorical variables. Outcomes and survivorship were analyzed for the entire study cohort. Continuous variables were compared using *t*-tests, while categorical variables were analyzed using Chi-square tests or Fisher’s exact tests when expected cell counts were less than five. Two-year implant survivorship was estimated using Kaplan–Meier analysis and truncated at 730 days to standardize follow-up duration. Kaplan–Meier estimates provide the probability of implant survival at each time point, and 95% confidence intervals were calculated to quantify uncertainty in survival estimates. Propensity score-based overlap weighting was used to create a balanced pseudo-population for adjusted analyses, with weighted logistic regression used to evaluate two-year revision, a weighted Cox proportional hazards model used to assess time to revision, and linear regression for PROMs [28]. No a priori power analysis was performed given the fixed number of eligible patients available during the study period. Because implant selection was partially surgeon-dependent and many surgeons used one implant system, a surgeon-level stratified analysis was not performed given the very small number of revision events in the full cohort. Instead, we performed a sensitivity analysis among the four surgeons using both implanted systems to address surgeon-specific implant bias. All statistical analyses were performed using SPSS Version 23 (IBM Corp., Armonk, NY, USA).

## 3. Results

A total of 220 patients met inclusion criteria, with 110 receiving Implant A and 110 receiving Implant B. Baseline characteristics are summarized in Table 1. Intraoperative patellar resurfacing differed, with patients receiving Implant B having higher rates of patellar resurfacing than those receiving Implant A (*p* = 0.016). Median follow-up was 3.3 years (range, 1.5 to 4.0 years) for Implant A and 2.2 years (range, 1.5 to 5.0 years) for Implant B (*p* = 0.058).

Unadjusted clinical outcomes are presented in Table 2. Ninety-day readmission rates were the same in both groups (4/110 [3.6%] vs. 4/110 [3.6%], *p* = 1.000), with similar distributions of orthopedic and non-orthopedic causes. 

**Figure 3:**
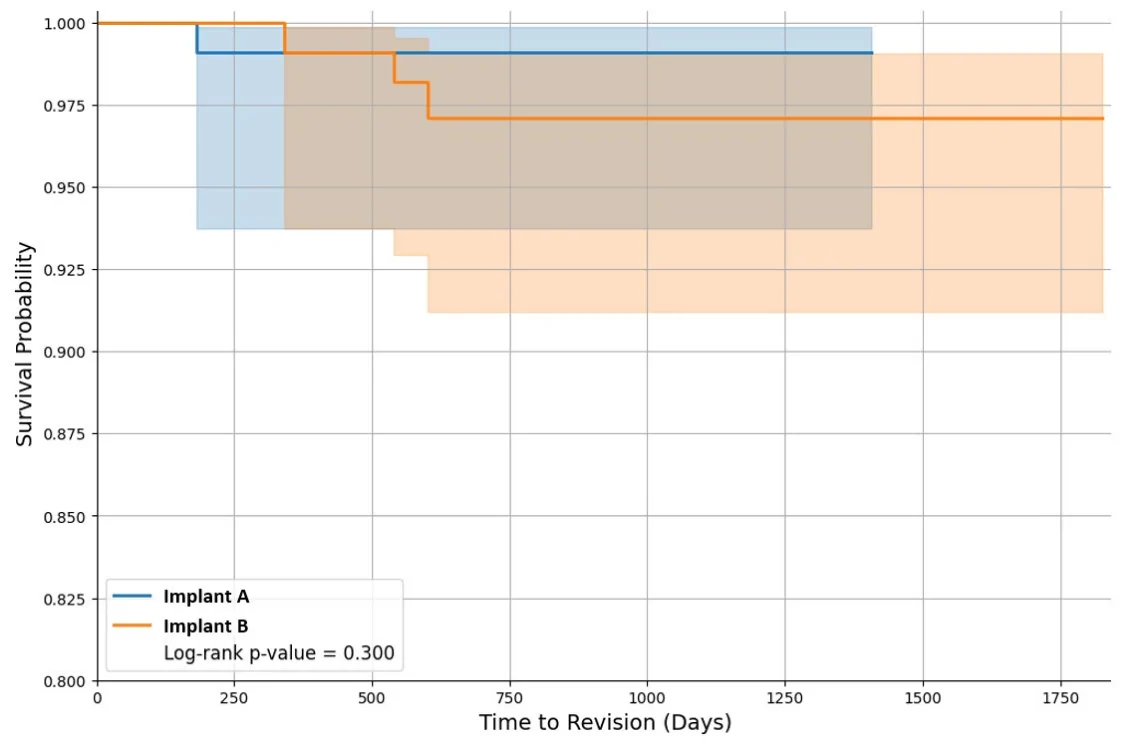
Kaplan–Meier curve for implant survivorship. Kaplan–Meier curve for survivorship of Implant A (blue) vs. Implant B (orange). Survivorship is defined as time without revision of the index components. Patella resurfacings in patients who had previously unresurfaced patellae are not counted as revisions for this analysis.

Revision of the femoral or tibial components occurred in one patient (0.9%) in the Implant A cohort and three patients (2.7%) in the Implant B cohort (*p* = 0.622). The one revision in the Implant A cohort was for anterior knee pain; one revision in the Implant B cohort was for periprosthetic joint infection, and the other two revisions were aseptic. Reoperations not involving femoral or tibial component revision occurred in five patients (4.5%) in the Implant A cohort and zero patients in the Implant B cohort (*p* = 0.060), including primary patellar resurfacing and arthroscopy for lysis of adhesions. Manipulation under anesthesia was performed in five patients (4.5%) with Implant A and five patients (4.5%) with Implant B (*p* = 1.000). Kaplan–Meier analysis (Figure 3) demonstrated high survivorship for both implants over two years, without a statistically significant difference in survival free from revision (log-rank *p* = 0.300).

Patient-reported outcomes are shown in Table 3. Preoperative KOOS, JR scores did not significantly differ between groups. At two weeks and one year postoperatively, KOOS, JR scores and changes from baseline did not differ significantly between groups. At three months, KOOS, JR scores were significantly higher in the Implant B cohort (57.3 vs. 62.4, *p* = 0.049), while the change from baseline did not differ significantly (Δ three months 17.8 vs. 15.8, *p* = 0.562). At two years, KOOS, JR scores were higher in the Implant A cohort, with a greater change from baseline. However, only seven patients in the Implant B cohort had paired preoperative and two-year KOOS, JR scores available. Achievement of the KOOS, JR MCID at one year was similar between groups (73.2 vs. 64.2%, *p* = 0.277). 

**Figure 4:**
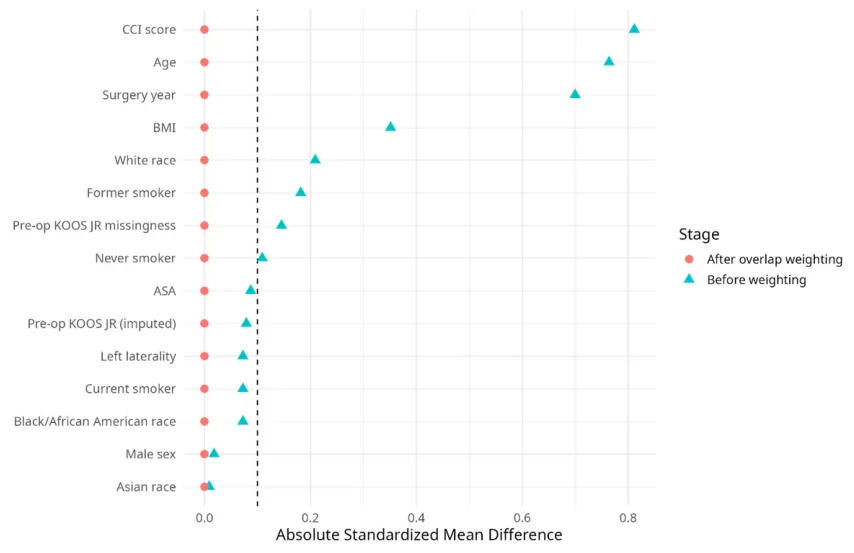
Covariate balance before and after overlap weighting, A vs. B. The dashed vertical line represents an absolute standardized mean difference of 0.1. CCI, Charlson Comorbidity Index; BMI, body mass index; KOOS JR, Knee Injury and Osteoarthritis Outcome Score for Joint Replacement; ASA, American Society of Anesthesiologists status.

Propensity score-based overlap weighting achieved balance across all measured baseline covariates (Figure 4).

In adjusted analyses of implants B and A, there was no statistically significant difference in two-year revision (weighted logistic regression: OR 2.17, 95% CI 0.10 to 157, *p* = 0.621) or in time to revision (weighted Cox proportional hazards model: HR 2.22, 95% CI 0.18 to 27.09, *p* = 0.533); these analyses were imprecise due to sparse events. Unadjusted rates of 90-day readmission, reoperation, and manipulation under anesthesia were similar between groups, and adjusted analyses did not demonstrate statistically significant differences for these outcomes. After adjustment, there was no significant difference between implant type and one-year KOOS, JR scores (*p* = 0.424), but there was a difference at two years, with Implant A resulting in an 18.3-point increase compared to Implant B (Table 4, 95% CI, 5.8 to 30.7, *p* = 0.005). This difference was likely unstable due to only seven patients having available changes in KOOS, JR scores at two years.

Among the four surgeons who used both implant systems, there were 59 patients, of whom 43 received Implant A and 16 received Implant B (Table 5). There were no revisions in either cohort. However, there were three readmissions in the Implant A cohort and zero within the Implant B cohort (*p* = 0.556). Reoperation occurred in four Implant A cases and zero Implant B cases (*p* = 0.555); of the reoperations, two were knee arthroscopies and two involved patellar resurfacing. These findings should be interpreted cautiously given the small sample sizes and sparse events. 

## 4. Discussion

In this retrospective comparative study of two cementless cruciate-retaining TKA designs, there were low revision rates in both implant groups, and no statistically significant difference in revision risk was detected between systems after adjustment with propensity score-based overlap weighting. However, these analyses were limited by the very small number of revision events and resulting imprecision. These findings add to a growing body of evidence suggesting that modern cementless TKA designs can achieve outcomes comparable to cemented fixation, showing benefits of cementless fixation for appropriately selected patients [5,11,16,17,18,19,20,21,22]. 

Prior registry and cohort analyses have demonstrated excellent mid-term survivorship for contemporary cementless implants [17,18,19,20,21,22], and randomized and comparative studies have reported similar or noninferior performance relative to cemented fixation in terms of survivorship, complications, and functional outcomes [6,8,9,10,17,18,19,20,21]. Our results corroborate these reports by showing that two different porous cruciate-retaining systems from separate manufacturers can both achieve favorable survivorship and patient-reported outcomes without clear evidence of superiority of one design over the other. This is consistent with prior work highlighting the importance of advances in porous coatings, surface topography, and implant design in overcoming the limitations of earlier cementless TKAs [11,12,13,14,15,16]. 

Although these two implants both include porous coatings, their tibial components employ different designs to achieve mechanical stability. Implant A uses a medialized keel along with anti-rotation press-fit features designed to engage dense medial bone, thereby resisting rotational forces and promoting early stability. By contrast, Implant B uses a central keel with four peripheral cruciform pegs that provide multiple points of fixation, aiming to improve initial press-fit stability and resist micromotion. These differences in fixation geometry may affect both rotational and translational stability, load transfer, and micromotion at the bone–implant interface. Because successful osseointegration depends on maintaining adequate initial stability in the early postoperative period, these mechanical differences could subsequently influence bone ingrowth, long-term tibial fixation, and implant survivorship. Despite these different fixation strategies, the lack of significant differences in outcomes in our cohort suggests that both designs may provide sufficient early stability to support similarly favorable clinical outcomes over the observed follow-up period. However, these clinical findings should not be interpreted as demonstrating biomechanical equivalence, as we did not specifically evaluate implant fixation or osseointegration.

While individual studies have reported differences in aseptic revision, the overall literature generally describes similar survivorship and outcomes in comparisons of cemented and porous cementless TKAs. For example, Fokter et al. compared survivorship of two-pegged porous cementless TKAs to cemented TKAs using the same implant systems in a retrospective study of 1,202 TKAs. They found similar 7-year all-cause survivorship (cementless vs. cemented: 97.4 vs. 97.8%, *p* = 0.63), but lower 7-year aseptic survivorship (97.9 vs. 99.4%, *p* < 0.05), and higher revision rates for aseptic indications among cementless TKAs (1.86 vs. 0.84%, *p* < 0.05) [29]. Other retrospective literature has generally shown no differences in outcomes between cementless and cemented TKAs. Sohn et al. found no differences in postoperative blood loss or transfusion rates among a cohort of 54 cementless and 53 cemented TKAs [30]. Additionally, Sheridan et al. compared 106 cemented and 152 cementless TKAs with a minimum two-year follow-up. They observed zero revisions among cementless and two revisions among cemented TKAs, finding no overall differences in all-cause revision rates (*p* = 0.17) [31]. Pasqualini et al. conducted a prospective, non-randomized study of 453 patients comparing fully cementless Triathlon constructs with cemented Triathlon constructs, and finding similar rates of adverse events and implant survivorship between groups (cementless vs. cemented: 98.2 vs. 97.5%, *p* = 0.44) [32]. A meta-analysis by Li et al., which included nine studies comparing porous tantalum cementless and cemented tibial components, similarly found no significant difference in implant revision rates [33]. However, the cemented group demonstrated a greater prevalence of radiolucent lines beneath the tibial component, suggesting differences in the local bone–implant environment despite similar clinical outcomes. Our findings are broadly consistent with this literature demonstrating high early survivorship of contemporary cementless fixation and extend the literature by directly comparing two cementless TKA designs with differing fixation geometries. However, we were limited by the low number of revision events, short follow-up, lack of radiographic analysis, and the absence of a cemented TKA comparison group.

In our cohort, Implant A demonstrated greater absolute improvement in KOOS, JR scores at two years, whereas Implant B showed somewhat higher scores at three months but a smaller overall gain by two years. Multiple factors could influence these trajectories, including baseline differences in age, BMI, comorbidities, activity expectations, surgeon-specific rehabilitation protocols, and time-varying follow-up and response patterns for KOOS, JR. Furthermore, at two years, only seven patients in the Implant B group had available information on the change in KOOS, JR from preoperative to postoperative follow-up, which is insufficient to support reliable statistical comparisons; these results should be interpreted with caution, and the reported *p*-values are likely unstable. From a mechanistic standpoint, cementless fixation may support durable pain relief and functional improvement over time by promoting osseointegration and potentially reducing micromotion at the bone–implant interface, which has a particular benefit in younger and/or higher-BMI patients who place greater mechanical demands on the construct [5,11,16,17,18,19,20,21,22]. 

This study has certain limitations. Its retrospective design is subject to selection bias and residual confounding, including surgeon choice of implant based on patient or anatomic factors that may not be fully captured in the available data. To address surgeon-selection bias, we performed a sensitivity analysis restricted to the four surgeons who used both implant systems; within this subgroup, no revisions occurred in either group and no statistically significant differences were observed in readmission or reoperation. However, this restricted analysis remained limited by the small subgroup size and sparse events and therefore could not fully account for surgeon-level confounding. Rates of intraoperative patellar resurfacing were also higher in Implant B, which could influence patient perceptions of pain and KOOS, JR scores. Additionally, KOOS, JR data were incompletely observed at multiple time points, and missing data may not have been completely random; this could bias estimates of functional trajectories. Although all patients met the minimum follow-up requirement for inclusion, we could not reliably determine interval-specific clinical follow-up at each postoperative time point and therefore could not fully assess attrition bias related to missing PROM data. No formal power analysis was performed due to the fixed number of patients enrolled in this study, and the study may have been underpowered to detect rare events such as revision. Surgeons demonstrated preferences for specific implant systems, and operations were performed across different calendar years; these factors may reflect differences in surgeon experience, learning curves, or perioperative protocols that are not fully accounted for, even with overlap weighting and covariate adjustment. Tourniquet use was not standardized across surgeons and may have varied in both application and duration. Given the 16 surgeons included in this study, this variability may have contributed to residual confounding between patients that could not be fully accounted for in the present analysis. Coronal plane alignment of the knee was also not measured routinely; therefore, we could not assess if native coronal alignment differed between groups or whether it contributed to residual confounding. Finally, due to the cohort size, the absolute number of revision events was small, which supports the continued usage of both implants but results in wide confidence intervals around hazard and odds ratio estimates, limiting the precision with which differences in survivorship could be estimated.

## 5. Conclusions

This study suggests that two widely used contemporary cementless cruciate-retaining TKA designs demonstrated low early revision rates and favorable short-term outcomes at two years despite employing different tibial porous fixation strategies. No statistically significant difference in revision risk was detected between implants; however, revision events were rare and estimates were imprecise. Together with existing registry, randomized, and observational data, these cementless TKA designs demonstrate promising short-term performance and safety profiles. Longer-term follow-up and larger multicenter studies may help determine whether any clinically meaningful performance differences emerge over time.

## Figures and Tables

**Table 1:** Baseline patient characteristics.

Demographic Characteristic	Implant A (*n* = 110)	Implant B (*n* = 110)	*p*-Values
Age, Mean (range)	57.7 (24 to 74)	63.2 (41 to 85)	<0.001
Sex, *n* (%)			0.845
Women	66 (60.0)	68 (61.8)	
Men	44 (40.0)	42 (38.2)	
Race, *n* (%)			0.490
White	53 (48.2)	69 (62.7)	
Black	32 (29.1)	24 (21.8)	
Asian	5 (4.5)	4 (3.6)	
Other/Unknown	20 (18.2)	13 (11.8)	
BMI, Mean (range)	34.3 (21.1 to 58.4)	32.2 (21.0 to 50.0)	0.011
Smoking Status, *n* (%)			0.050
Never	78 (70.9)	66 (60.0)	
Former	21 (19.1)	41 (37.3)	
Current	11 (10.0)	3 (2.7)	
ASA Status			0.464
1	3 (2.7)	3 (2.7)	
2	73 (66.4)	65 (59.1)	
3	31 (28.2)	42 (38.2)	
4	3 (2.7)	0 (0.0)	
Patella Resurfacing, *n* (%)	64 (58.2)	81 (73.6)	0.016
Median Follow-up, Years (Range)	3.3 (1.5 to 4.0)	2.2 (1.5 to 5.0)	0.058

n, number of; %, percent; BMI, body mass index; ASA, American Society of Anesthesiologists.

**Table 2:** Clinical outcomes at latest follow-up, unadjusted.

Clinical Outcomes at Latest Follow-Up	Implant A (*n* = 110)	Implant B (*n* = 110)	*p*-Values
90-day Readmission, *n* (%)	4 (3.6)	4 (3.6)	1.000
Orthopedic Cause	1 (0.9)	2 (1.8)	
Non-Orthopedic Cause	3 (2.7)	2 (1.8)	
Revision, *n* (%)	1 (0.9)	3 (2.7)	0.622
PJI	0 (0)	1 (0.9)	
Aseptic	1 (0.9)	2 (1.8)	
Reoperation, *n* (%)	5 (4.5)	0 (0)	0.060
Patellar Resurfacing for Previously Unresurfaced Knees *	2 (4.3)	0 (0)	0.520
Arthroscopy	3 (2.7)	0 (0)	0.246
MUA, *n* (%)	5 (4.5)	5 (4.5)	1.000

PJI, periprosthetic joint infection; MUA, manipulation under anesthesia; n, number of; %, percent. * Note: 46 patients who had Implant A and 29 patients who had Implant B had unresurfaced knees.

**Table 3:** Patient-reported outcomes.

Outcome Metric	Implant A (*n* = 110)	Implant B (*n* = 110)	*p*-Values
KOOS, JR, mean (range), *n*			
Preoperative	40.3 (0 to 71.0), 81	43.8 (3.8 to 77.7), 64	0.155
2 weeks postop	52.4 (25.0 to 80.0), 52	54.9 (28.0 to 76.0), 52	0.316
Δ 2 week	12.1 (−40.0 to 76.0), 45	8.9 (−17.5 to 45.0), 46	0.367
3 months postop	57.3 (25.0 to 85.0), 43	62.4 (42.0 to 92.0), 46	0.049
Δ 3 months	17.8 (−17.0 to 50.0), 39	15.8 (−9.7 to 46.3), 39	0.562
1 year postop	64.6 (34.0 to 100.0), 38	67.6 (42.5 to 100.0), 36	0.429
Δ 1 year	26.7 (2.5 to 60.0), 33	19.9 (−7.5 to 43.0), 25	0.095
2 years postop	73.2 (34.2 to 100.0), 73	54.3 (10.3 to 100.0), 11	0.006
Δ 2 years	32.5 (−15.8 to 100.0), 55	11.2 (−16.0 to 48.0), 7	0.026
Achievement of KOOS, JR MCID at 1 year, ratio (%)	52/71 (73.2)	34/53 (64.2)	0.277

KOOS, JR, Knee injury and Osteoarthritis Outcome Score for Joint Replacement; n, number of; postop, postoperative; Δ, change from preoperative, MCID, minimally clinically important difference (14 at one year for KOOS, JR).

**Table 4:** Adjusted outcomes after overlap weighting.

Outcome, Implant B vs. Implant A	Adjusted Estimate	95% CI	*p*-Values
2-Year Revision, Logistic Regression	OR 2.17	0.10 to 157	0.621
Time to Revision, Cox Model	HR 2.22	0.18 to 27.09	0.533
1-Year KOOS, JR, Linear Regression	Difference, B − A: -2.5	−8.7 to 3.7	0.424
2-Year KOOS, JR, Linear Regression	Difference, B − A: −18.3	−30.7 to −5.8	0.005

CI, confidence interval; KOOS, JR, Knee injury and Osteoarthritis Outcome Score for Joint Replacement; OR, odds ratio; HR, hazard ratio.

**Table 5:** Sensitivity analysis among four surgeons using both implant systems.

Outcome	Implant A (*n* = 43)	Implant B (*n* = 16)	*p*-Values
Revision, *n* (%)	0 (0.0)	0 (0.0)	NA
90-Day Readmission, *n* (%)	3 (7.0)	0 (0.0)	0.556
Reoperation, *n* (%)	4 (9.3)	0 (0.0)	0.555

n, number of.

## Data Availability

The original contributions presented in this study are included in the article. Further inquiries can be directed to the corresponding authors.

## Abbreviations

The following abbreviations are used in this manuscript:

ASA	American Society of Anesthesiologists
BMI	Body mass index
CCI	Charlson Comorbidity Index
Implant A	LEGION CONCELOC Knee System
Implant B	Triathlon Cementless Knee System
KOOS, JR	Knee Injury and Osteoarthritis Outcome Score for Joint Replacement
LOS	Length of stay
MCID	Minimum clinically important difference
MUA	Manipulation under anesthesia
PROMs	Patient-reported outcomes
TKA	Total knee arthroplasty
